# Patient and provider interventions for managing osteoarthritis in primary care: protocols for two randomized controlled trials

**DOI:** 10.1186/1471-2474-13-60

**Published:** 2012-04-24

**Authors:** Kelli D Allen, Hayden B Bosworth, Dorothea S Brock, Jennifer G Chapman, Ranee Chatterjee, Cynthia J Coffman, Santanu K Datta, Rowena J Dolor, Amy S Jeffreys, Karen A Juntilla, Jennifer Kruszewski, Laurie E Marbrey, Jennifer McDuffie, Eugene Z Oddone, Nina Sperber, Mary P Sochacki, Catherine Stanwyck, Jennifer L Strauss, William S Yancy Jr

**Affiliations:** 1 Health Services Research and Development Service, Durham VA Medical Center, Durham, NC, USA; 2 Department of Medicine, Duke University Medical Center, Durham, NC, USA; 3 Center for Aging and Human Development, Duke University, Durham, NC, USA; 4 Department of Psychiatry and Behavioral Science, Duke University, Durham, NC, USA; 5 Department of Biostatistics and Bioinformatics, Duke University, Durham, NC, USA; 6 HSR&D (152), VA Medical Center, 508 Fulton Street, Durham, NC, 27705, USA; 7 Division of General Internal Medicine, Duke University Medical Center, 411 West Chapel Hill Street Suite 500, Durham, NC, 27701, USA

**Keywords:** Osteoarthritis, Physical activity, Weight reduction program, Pain management, Intervention study

## Abstract

**Background:**

Osteoarthritis (OA) of the hip and knee are among the most common chronic conditions, resulting in substantial pain and functional limitations. Adequate management of OA requires a combination of medical and behavioral strategies. However, some recommended therapies are under-utilized in clinical settings, and the majority of patients with hip and knee OA are overweight and physically inactive. Consequently, interventions at the provider-level and patient-level both have potential for improving outcomes. This manuscript describes two ongoing randomized clinical trials being conducted in two different health care systems, examining patient-based and provider-based interventions for managing hip and knee OA in primary care.

**Methods / Design:**

One study is being conducted within the Department of Veterans Affairs (VA) health care system and will compare a Combined Patient and Provider intervention relative to usual care among n = 300 patients (10 from each of 30 primary care providers). Another study is being conducted within the Duke Primary Care Research Consortium and will compare Patient Only, Provider Only, and Combined (Patient + Provider) interventions relative to usual care among n = 560 patients across 10 clinics. Participants in these studies have clinical and / or radiographic evidence of hip or knee osteoarthritis, are overweight, and do not meet current physical activity guidelines. The 12-month, telephone-based patient intervention focuses on physical activity, weight management, and cognitive behavioral pain management. The provider intervention involves provision of patient-specific recommendations for care (e.g., referral to physical therapy, knee brace, joint injection), based on evidence-based guidelines. Outcomes are collected at baseline, 6-months, and 12-months. The primary outcome is the Western Ontario and McMasters Universities Osteoarthritis Index (self-reported pain, stiffness, and function), and secondary outcomes are the Short Physical Performance Test Protocol (objective physical function) and the Patient Health Questionnaire-8 (depressive symptoms). Cost effectiveness of the interventions will also be assessed.

**Discussion:**

Results of these two studies will further our understanding of the most effective strategies for improving hip and knee OA outcomes in primary care settings.

**Trial registration:**

NCT01130740 (VA); NCT 01435109 (NIH)

## Background

Osteoarthritis (OA) is one of the most common chronic health conditions and a leading cause of pain and disability among adults [1-3]. The hip and knee are two commonly affected joints [4,5], having a significant impact on walking and other daily activities. The prevalence of OA is on the rise, and this trend is expected to continue [6]. For example, recent data from the Framingham Osteoarthritis Study show that over about the past 20 years, the prevalence of knee OA approximately tripled in men and almost doubled in women [7]. Therefore, in addition to the substantial toll of OA at the individual level [8], this health problem has a significant societal impact due to health care costs [9,10].

Evidence-based guidelines emphasize that adequate management of hip and knee OA requires a combination of behavioral and medical strategies [11-14]. Physical activity and weight management are two key behavioral strategies for managing hip and knee OA, supported by numerous clinical trials and emphasized in treatment guidelines. However, the majority of adults with OA are physically inactive and / or overweight. For example, among patients with knee OA in the Osteoarthritis Initiative, only about 13% of men and 8% of women met the 2008 Department of Health and Human Services Physical Activity Guidelines regarding aerobic activity [15]. Data from the Centers for Disease Control and Prevention show that 66% of US adults with arthritis (primarily OA) are overweight or obese [16]. These data clearly show that efforts are needed to improve physical activity and healthy eating behaviors among individuals with OA. Cognitive behavioral approaches to pain management can also improve outcomes among patients with OA [17], but programs to teach these skills are also not widely accessible to patients.

Although there is a strong evidence base for many clinical strategies for managing OA (e.g., joint injections, physical therapy, pain medications), some of these treatments are under-utilized. Several studies have shown low “pass rates” for quality indicators of care for OA, including assessment of pain and function, referrals to other providers (when indicated), appropriate prescribing of pain medications, and recommendations for exercise and weight management [18-20]. Only a few studies have examined provider-based interventions to enhance management of OA in clinic settings [21-23]. These studies have shown improvements in OA treatment practices and some patient outcomes following the provider interventions. However, these programs were time-intensive (making them infeasible in most real-world clinical settings) and only reached a small number of providers. There is still a need to develop and test provider-based interventions that can be practically disseminated in real-world clinical settings to help facilitate improvements in care for patients with OA.

This manuscript describes two ongoing clinical trials in two different health care systems that are examining patient-based and provider-based interventions for managing hip and knee OA in primary care. The patient-based intervention focuses on physical activity, weight management, and cognitive behavioral pain management, and the provider intervention involves provision of patient-specific recommendations for care, based on evidence-based guidelines. Through these two studies, we are able to evaluate these interventions in distinctly different health care settings and patient populations. One study is being conducted within the Department of Veterans Affairs (VA) health care system and will compare a combined patient-based and provider-based intervention relative to usual care. Another study is being conducted within the Duke Primary Care Research Consortium and will compare patient-based, provider-based, and combination (patient + provider) interventions relative to usual care. We are reporting the methods for these two trials together because they involve common interventions and measures; design issues that differ between the studies are specified when appropriate.

### Hypotheses

Hypotheses for the VA-based study (reflecting a 2-arm trial) are:

Primary

H_1_: Patients with hip and/or knee OA who receive a comprehensive intervention (including both patient-based and provider-based components) will have a larger, clinically relevant improvement in self-reported pain, stiffness and function, as measured by the Western Ontario and McMasters Universities Osteoarthritis Index (WOMAC), compared with usual care.

Secondary

H_2_: The comprehensive OA intervention will result in improvement in objectively assessed physical function (using the Short Physical Performance Test Protocol) when compared to usual care.

H_3_: The comprehensive OA intervention will result in improvement in depressive symptoms (measured with the Patient Health Questionnaire-8 (PHQ-8) [24]) when compared to usual care.

Hypotheses for the Duke-based study (reflecting a 4-arm trial) are:

Primary

H_1_: Compared to usual care, patients with hip and/or knee OA who receive a combined patient-based and provider-based intervention will have a larger improvement in self-reported pain, stiffness and function, as measured by the WOMAC, than either a provider-based or patient-based intervention alone.

H_2_: Patients with hip and/or knee OA who receive either the patient-based OR provider-based intervention will have clinically relevant improvements in WOMAC scores when compared to usual care.

Secondary

H_3_: The patient-based, provider-based, and combined interventions will each result in improvement in objectively assessed physical function (using the Short Physical Performance Test Protocol) when compared to usual care, and the combined intervention will result in the greatest improvement.

H_4_: The patient-based, provider-based, and combined interventions will each result in improvement in depressive symptoms (measured with the PHQ-8 [24]) when compared to usual care, and the combined intervention will result in the greatest improvement.

## Methods / Design

### Trial design

Both the VA-based and Duke-based studies are randomized controlled trials, but the study designs differ due to variations in the arms being tested. The VA-based study is a cluster randomized controlled trial, in which Primary Care Providers (PCPs) are assigned to one of two study arms: OA Intervention and Usual Care Control. PCPs assigned to the OA Intervention receive the Provider Intervention described below, and their enrolled patients receive the Patient Intervention, also described below.

**Figure 1:**
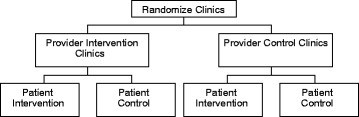
Duke study design.

Figure 1 shows the design of the Duke-based trial, in which clinics are randomized to Provider Intervention vs. Control, then patients within those clinics are assigned to Patient Intervention vs. Control. This results in patients being assigned to one of four study arms: 1.) Patient Intervention Only, 2.) Provider Intervention Only, 3.) Patient Intervention + Provider Intervention and, 4.) Usual Care Control. For both VA and Duke studies, all participants continue with any other usual medical care they receive for OA. After completion of follow-up assessments, participants assigned to the Patient Control condition are given the materials for the Patient Intervention, and clinics / PCPs who were assigned to the Provider Control condition are given the patient-specific recommendations for the Provider Intervention. These studies were reviewed and approved by the Durham VA Medical Center and Duke Institutional Review Boards, respectively.

### Study settings

The VA-based study is being conducted among patients at the Durham VA Medical Center and its associated community-based outpatient clinics. The Durham VA Medical Center serves about 53,600 veterans, about 68% of whom are age 55 or older and therefore in the prime age category for the development of OA. The Duke-based study is being conducted within the Duke Primary Care Research Consortium, a primary care-based research network composed of 30 practices in 8 counties of the Piedmont area of North Carolina (both urban and rural).

### Participants

Both patients and PCPs serve as participants in these studies. In the VA-based study, we are enrolling approximately 30 PCPs and 10 patients (5 white, 5 non-white) per PCP, for a total sample size of n = 300. In the Duke-based study, we are enrolling PCPs from 10 PCRC clinics and approximately 56 patients per clinic, for a total sample size of n = 560.

All participants must have hip OA (based on radiographic evidence in the electronic medical record) and / or knee OA (based on radiographic evidence in the electronic medical record OR meeting American College of Rheumatology clinical criteria [25]). Participants must also have current symptoms in the joint(s) with OA. Specifically, participants must answer “yes” to two questions: “In the past 12 months, have you had pain, aching, stiffness, or swelling in or around a hip or knee joint with arthritis?” and “Were these symptoms present on most days for the past month?” [26]. Because the Patient Intervention for this study focuses on weight management and physical activity, participants must also be overweight (body mass index (BMI) ≥ 25) and not currently meeting weekly physical activity guidelines set forth by the Departments of Health and Human Services (2 hours and 30 minutes of moderate intensity or 1 hour and 15 minutes of vigorous intensity aerobic activity plus 2 or more sessions of muscle strengthening exercises) [27]. Exclusion criteria for the study are:

· Rheumatoid arthritis, fibromyalgia, or other systemic rheumatic disease

· History of gout in knee or hip

· Total joint replacement (knee or hip) surgery, other knee or hip surgery, meniscus tear, or ACL tear in the past 6 months

· On waiting list for / planning arthroplasty

· Hospitalized for a stroke, myocardial infarction, heart failure, or coronary artery revascularization in the past 3 months

· Motor neuron diseases, Parkinson’s disease, multiple sclerosis, Paget’s disease

· Quadriplegic or paraplegic

· Dementia or other memory loss condition

· Metastatic cancer

· Referral for Hospice or Palliative Care

· Nursing home resident

· Active diagnosis of psychosis

· Serious personality disorder

· Current, uncontrolled substance abuse disorder

· Severely impaired hearing or speech (patients must be able to respond to phone calls)

· Blindness

· No access to a telephone

· Inability to understand or speak English

· Participating in another OA intervention or other lifestyle change study

· Females: currently pregnant or planning to become pregnant

· Have not seen PCP in the past 12 months (VA) or 18 months (Duke)

· Other health condition or personal issue judged by a study team member or primary care physician to make the patient inappropriate for study participation

· Other self-reported medical problem that would prohibit participation in the study

### Recruitment and enrollment

Recruitment and enrollment procedures are very similar for the VA-based and Duke-based studies. We first identify patients of participating PCPs / clinics who have diagnoses of hip and / or knee OA, based on relevant ICD-9 codes from electronic medical records (including 715.00, 715.09, 715.10, 715.15, 715.16, 715.25, 715.26, 715.30, 715.35, 715.36, 715.80, 715.89, 715.90, 715.95, 715.96, 719.40, 719.45, 719.46, 719.49). We then further examine the medical record to confirm the presence of an OA diagnosis and scan for exclusion criteria. We mail introductory letters to patients who meet criteria based on electronic medical records, on behalf of their PCP. This is followed about 1–2 weeks later by a screening telephone call to further assess eligibility, with particular focus on criteria that may not appear in the electronic medical record. Eligible, interested patients are asked to meet a study team member at the clinic site to complete the consent process and baseline questionnaires. Immediately following the consent process, we assess clinical criteria for knee OA [25], measure height and weight to determine BMI, and administer several questions about pain and physical function. If patients are not overweight according to BMI criteria, do not meet clinical criteria for knee OA (and also do not have radiographic evidence of hip or knee OA in at least one joint), or do not meet pain criteria, they are excluded from the study. Following the initial visit, participants are asked to complete a Food Frequency Questionnaire (FFQ) at home and return it via mail within one week. Participants are called with their randomization assignment once the FFQ has been received by the study team (or after about 2 weeks if it has not been returned and is therefore considered missing data).

### Randomization

Randomization for both studies is based on a computer generated sequence maintained by the project statistician. For the VA-based study, we stratify randomization of providers according to high vs. low volume of female patients (<15% vs. ≥15%), to ensure the groups are balanced in this respect. For the Duke-based study, clinics are randomized in pairs that are selected based on common characteristics such as approximate patient panel size and PC specialty (Family Medicine vs. Internal Medicine). For both studies, patient randomization is stratified according to race (white / non-white), and the Duke-based study is also stratified by patient gender. This is important because there are known differences in OA-related pain and function according to both gender and race, and it is possible that intervention effects may differ across these demographic variables as well.

### Interventions

#### Patient behavioral intervention for OA

This is a twelve-month intervention that includes the following elements: monthly telephone calls by a counselor to support behavior change, written patient educational materials, an exercise video for patients with OA, and a CD of relaxation exercises (each described below). This intervention is grounded in social cognitive theory, focusing on five determinants of health behavior change: self efficacy, knowledge of health risks and practices, outcome expectations regarding the costs and benefits of health behaviors, health goals, and addressing perceived barriers and facilitators of health [28].

##### Telephone Calls

Telephone calls are the core component of this intervention. Individuals with training in counseling and / or health education and behavior change deliver this intervention, with oversight provided by study co-investigators who have experience in each substantive aspect of the program (e.g., physical activity, weight management, and cognitive behavioral strategies). A summary of activities for all calls is shown in Table 1. Calls are scheduled twice per month for the first six months, then monthly for the last six months. During the first six months, the first scheduled call is a “content” call (e.g., new educational information is reviewed), and the second call is used to review participants’ progress toward goals. All calls use standardized scripts to assist with consistency of information delivery.

**Table 1:** Summary of activities for patient intervention calls

**Month #**	**Call #**	**Activities / Modules**
**1**	**1**	- Introduction to Intervention and Materials
		- Introduction to Osteoarthritis
		- Information Session #1 for Physical Activity or Healthy Eating (Participant Choice)
		- Cognitive or Behavioral Strategy – Activity Pacing Discussion #1
		- Goal Setting
	**2**	- Goal Review
**2**	**1**	- Information Session #2 for Physical Activity or Healthy Eating
		- Cognitive or Behavioral Strategy – Activity Pacing Discussion #2
		- Goal Setting
	**2**	- Goal Review
**3**	**1**	- Information Session #3 for Physical Activity or Healthy Eating
		- Cognitive or Behavioral Strategy – Breathing Relaxation Discussion #1
		- Goal Setting
	**2**	- Goal Review
**4**	**1**	- Information Session #1 for Physical Activity or Healthy Eating (Whichever not discussed in months 1–3)
		- Cognitive or Behavioral Strategy – Breathing Relaxation Discussion #2
		- Goal Setting
	**2**	- Goal Review
**5**	**1**	- Information Session #2 for Physical Activity or Healthy Eating
		- Cognitive or Behavioral Strategy – Distraction Discussion #1
		- Goal Setting
	**2**	- Goal Review
**6**	**1**	- Information Session #2 for Physical Activity or Healthy Eating
		- Cognitive or Behavioral Strategy – Distraction Discussion #2
		- Goal Setting
	**2**	- Goal Review
**7**	**1**	- Cognitive or Behavioral Strategy – Progressive Muscle Relaxation Discussion #1
		- Goal Setting
**8**	**1**	- Cognitive or Behavioral Strategy – Progressive Muscle Relaxation Discussion #2
		- Goal Setting
**9**	**1**	- Cognitive or Behavioral Strategy – Cognitive Restructuring Discussion #1
		- Goal Setting
**10**	**1**	- Cognitive or Behavioral Strategy – Cognitive Restructuring Discussion #2
		- Goal Setting
**11**	**1**	- Make-Up or Review
		- Goal Setting
**12**	**1**	- Make-Up or Review
		- Goal Setting

During the first three months of the study, participants are asked to choose whether to focus on either weight management or physical activity. Both the educational content and the goal-setting focus on that topic. At the first call for each of the first three months, the counselors provide a summary of key points that will be covered related to physical activity or weight management content (listed below).

#### Physical activity

Month 1: General Information about Physical Activity and OA & Aerobic Activity; Managing Pain with Physical Activity

Month 2: Stretching Exercises; General Tips for Success with Physical Activity

Month 3: Strengthening Exercises

#### Weight management

Month 1: Setting a Goal Weight & Eating Three Small Meals Per Day

Month 2: Control Your Calories

Month 3: Think Healthily about Food, and Involve Others in Your Weight Management Efforts

During the second three months of the study, the call content and goals focus on the other topic (weight management or physical activity). However, participants may also continue with goals they had previously been working toward during the first three months of the study.

In addition to the educational content provided, goal setting and / or review (for physical activity and / or weight management) is conducted during each telephone call. During the first phone call, participants are guided in the process of selecting a broad goal related to either physical activity or weight management. While patients are enabled to choose their own goals, the counselors provide advisement about goals that are associated with clinically relevant changes in health outcomes. With regard to physical activity, we encourage participants to work toward completing 2 hours and 30 minutes of moderate intensity aerobic exercise per week, 2 sessions of strengthening exercises per week, and stretching exercises daily as a long-term goal. These recommendations are based on U.S. physical activity guidelines [27]. However, counselors work with participants to determine reasonable short-term goals based on their pain and functional limitations. With regard to weight management, NIH guidelines recommend that patients who are overweight be encouraged to lose 10% of initial body weight [29]. However, for some participants this may seem like a difficult initial goal. Prior research has shown that for adults with knee OA who are overweight, losing even 5% of body weight can produce clinically relevant improvements in pain and function [30]. Therefore the counselors work with each participant to choose a reasonable, individualized weight loss goal. Participants may continue with any goal for as long as they choose, and they may have more than one active goal.

During each phone call, participants are also guided in the process of selecting specific action plans toward meeting their goal(s). Action plans are written for each of the subsequent weeks between the current and next scheduled telephone call. As a part of developing action plans, the counselors ask participants to rate their self-efficacy for completing each action plan on a scale of 1 to 10. If participants rate their self-efficacy lower than 7, the counselors recommend they revise this plan so they are more confident they will be able to carry it out. Prior research has shown that a self-efficacy rating of 7 or above is associated with a greater likelihood of accomplishing the plan [31]. The counselors also ask participants about the action plans they have been working on since the prior call, including any barriers they encountered. The counselors guide participants in a process of problem-solving any barriers, and this is incorporated into the process of developing new action plans.

Cognitive behavioral strategies are also discussed during telephone calls according to the schedule shown in Table 1. We have chosen to include this component throughout the program because cognitive behavioral strategies (e.g. cognitive restructuring) can be helpful for not only pain control, but also for working toward behavioral goals such as healthy eating and physical activity. The specific topics included in the cognitive behavioral component of the intervention are activity pacing, breathing relaxation, distraction, progressive muscle relaxation, and cognitive restructuring. Each of these skills is discussed at two telephone calls. The first call involves education about the specific strategy (including a rationale for its application to the management of OA-related pain), basic instruction in the skill, and development of a plan for independent practice and application of the skill. During the second call for each skill, the participant and counselor work collaboratively to review progress towards mastering, adapting, and applying the skills to meet the participant’s individual goals. Motivational interviewing strategies are employed throughout the intervention to identify and address any ambivalence participants exhibit regarding goals or applying new skills [32,33]. These strategies include asking open ended questions, reflective listening, developing discrepancy, rolling with resistance and eliciting change talk.

##### Written Patient Educational Materials

We developed a low-literacy patient educational booklet that covers the following topics: 1. What Is Osteoarthritis?, 2.) Physical Activity and Osteoarthritis, 3. Weight Management, 4. Skills for Managing Pain (Cognitive Behavioral Strategies). Patients are asked to review these materials during the intervention period. The booklet also includes worksheets for documenting goals and action plans related to physical activity and weight management, as well as worksheets for documenting practice of cognitive behavioral skills.

##### Exercise Video

Participants are given a copy of an exercise video called *Take Control with Exercise*, created by the Arthritis Foundation. Participants are also given therapy bands, since these are used in some of the exercises on the video.

##### Relaxation CD

Breathing relaxation and progressive muscle relaxation are two skills covered in this intervention. Participants are given CDs with audio instructions to facilitate practice of these skills. These were developed specifically for this study.

#### Provider intervention

The Provider Intervention involves delivery of patient-specific recommendations, delivered at the point of care. Specific recommendations include the following: 

· Refer to physical therapist for evaluation and / or therapeutic exercises

· Refer for evaluation for knee brace

· Refer to weight management program

· Refer to physical activity program

· Perform or refer for intra-articular injection

· Recommend or prescribe Topical NSAID or Capsaicin

· Add gastroprotective agent or remove from NSAID (patient has risk factors for GI bleeding)

· Discuss discontinuation of OTC NSAID with patient (patient has risk factors for GI bleeding)

· Discuss the possibility of trying a new / alternate pain medication with patient

· Referral to orthopedic for evaluation for joint replacement surgery (if no contraindications)

These recommendations were selected on the basis of treatment guidelines for managing hip and knee OA [12-14,34]. For most of these treatments, there are not specific criteria (e.g., level of pain severity) to guide use. Therefore our study team and other content experts developed algorithms that guide when a treatment option would be reasonable for a PCP to consider for a given patient. Algorithms are shown in Additional file 1. Data that feed into these algorithms are derived from medical records and baseline assessments (including both objective measures and patient self-report measures). The study team monitors upcoming visits for all participants enrolled in the Provider Intervention, and recommendations are delivered to PCPs within about one week prior to participants’ first routine (non-urgent) visit after enrolling in the study. These recommendations are delivered to PCPs within the electronic medical record systems. Within the VA system, the study team provides PCPs with specific instructions for requesting consults for some of the recommendations, including knee braces (specific types); physical therapy visits, referral to the MOVE! weight management program, and orthopedic service visits for joint injections and evaluation for surgery. For each Duke clinic, the study team provides a list of local resources for physical activity and weight management programs to which PCPs may refer or direct patients.

### Measures

#### Overview and timing

For both VA-based and Duke-based studies, the primary time point for outcome assessment is 12-months following baseline assessment. Baseline and 12-month assessments are conducted in-person (except when transportation or time constraints require completion of portions of the questionnaire via telephone). We are also measuring the primary outcome (and a few other selected outcomes) via telephone at 6-months following baseline to examine the time trajectory of changes during the study period. For the Duke-based study, we will additionally evaluate the primary and several other outcomes at 18-months and 24-months following baseline, via telephone, to examine the sustainability of any intervention effects observed at the 12-month observation point.

#### Primary and secondary outcomes

The primary outcome measure for this study is the WOMAC, a measure of lower extremity pain (5 items), stiffness (2 items), and function (17 items). Secondary outcomes are objective physical function and depressive symptoms, as these are both key outcomes for patients with OA and are associated with pain. Objective physical function is being assessed with the Short Physical Performance Test Protocol [35], which is a series of five tests covering the domains of balance (3 tests), gait speed (8-foot walk), and time to rise from a chair and return to the seated position five times. Depressive symptoms are being assessed with the Patient Health Questionnaire (PHQ-8), a reliable and valid measure of depression [36].

#### Process / intermediate measures

Several process measures are being evaluated in order to describe changes in intermediate outcomes that may be associated with the interventions, either as mediating or moderating factors. Process measures include the Arthritis Self Efficacy [37], Self-Efficacy for Exercise scale [38], Perceived Competence for Maintaining a Healthy Diet [39], physical activity (Community Health Activities Model Program for Seniors; CHAMPS) [40,41], dietary intake using the Block Brief 2000 Food Frequency Questionnaire (3-Month Recall) [42], Pain Catastrophizing Scale [43], Stone and Neale’s Daily Coping Inventory adapted for pain coping [44], and the number of completed intervention calls (for those in the patient intervention groups).

#### OA-related treatments and medical care

We are asking patients to report their use of OA-related treatments, including: visits to physical therapists, visits to orthopedic surgeons, receipt of joint injections, use of knee braces, pain medication use, and topical analgesic use. These data will allow us to evaluate whether there are any differences in OA treatments (during the study period) between patients of providers / clinics who are in the Provider intervention vs. Provider control arms.

#### Participant characteristics

We are collecting patient demographic and clinical characteristics including: self-reported age, gender, race/ethnicity, household financial situation, education level, marital status, work status, health literacy (using the Rapid Estimate of Adult Literacy -SF) [45], body mass index, history of knee and hip injuries and surgeries, duration of OA symptoms, general self-rated health, smoking, alcohol use, and comorbid illnesses using the Self-Administered Comorbidity Questionnaire [46].

#### Exploratory measures

In addition, we are collecting exploratory measures that are of interest in this patient population and type of intervention. These measures include additional pain measures (visual analog and visual numeric scales [47,48], measure of pain predictability from the Measure of Constant and Intermittent Osteoarthritis Pain [49]), fatigue visual analog scale [48], sleep quality (Insomnia Severity Index [28] and Berlin Questionnaire [50]), foot symptoms (Foot Assessment Clinical Tool [51]), Social Support for Diet and Exercise [52], Satisfaction with Physical Function [53], and Global Assessment of Joint Symptom Change (at follow-up only [54,55]). At follow-up we will also assess perceptions of patients and providers about the respective interventions, as well as suggestions for improvements for future implementation, using open-ended questions.

### Sample size

For the VA-based study, we will enroll approximately 30 providers (15 per group) with 10 patients per provider (150 per group), for a total sample size of 300. Our goal was to have sufficient sample size to detect a moderate effect size of approximately 0.30 for the primary hypothesis with 80% power and a type-I error rate (alpha) of 0.05. Based on expected mean baseline WOMAC scores of 38 with a standard deviation of 14 (anticipated from our prior pilot work), this effect size translates to a 4.2 point difference at 12-months, which is equivalent to approximately 11% improvement from baseline scores. Sample size calculations were based on methods appropriate for ANCOVA type analyses [56] and adjusted for provider clustering using the method of Donnar & Klar [57]. A correlation of 0.60 between time points was estimated based on our pilot data, and we accounted for a 12% attrition rate. This yielded a final sample size of 150 participants per group.

For the Duke-based study, we will enroll approximately 56 participants across each of 10 clinics, for a total sample size of 560. Our goal was to have sufficient sample size to detect moderate effect size of approximately 0.39 for the primary hypotheses with 80% power and a type-I error rate (alpha) of 0.05. Based on the same pilot data described above, this effect size translates to a 5.5 point difference at 12-months which is equivalent to approximately 15% improvement from baseline scores. For H_1_, this implies that compared to usual care we can detect a 15% greater improvement in WOMAC scores for participants that receive the combined intervention than the improvement in WOMAC scores for participants that receive either the Patient or Provider only at 12-months. For H_2_, this implies we can detect a 15% greater improvement in those that receive either the Patient or Provider intervention as compared to usual care at 12-months. Sample size calculations were based on methods appropriate for ANCOVA type methods, [56] adjusted for clinic clustering using the method of Donnar & Klar [57]. A correlation of 0.60 between time points was estimated based on our pilot data, and we accounted for a 15% attrition rate. This yielded a final sample size of 140 participants per group.

### Data analyses

The main conclusions drawn from these trials will be based on the pre-specified primary and secondary hypotheses and will be tested with two-sided p-values at the standard 0.05 level. Primary analyses will be conducted on an intent-to-treat basis; participants will be analyzed in the group to which they were assigned, regardless of intervention adherence, using all data up to the 12-months follow-up or last available measurement prior to exclusion or dropout [58]. Statistical analyses will be performed using SAS for Windows (Version 9.2: SAS Institute, Cary, NC) and R (http://www.R-project.org).

For the VA-based study, our primary hypothesis will be tested using a hierarchical linear model with patients nested within providers; baseline, 6- and 12-month values in the response vector will be used to estimate changes in WOMAC scores over time and test the primary hypothesis [59]. A random effect will be included in the model to account for clustering of patients within providers, as the providers are the unit of randomization. Because of the small number of time points, we will apply an unstructured covariance matrix to take into account the within-patient correlation between repeated measures over time. The predictors in the model will include a dummy coded time effect and an indicator variable for the intervention interacting with the time effect. The fixed-effect portion of the model will have the form [60]

(1) $$Y_{\mathrm{ijk}}=\beta_{0}+\beta_{1}*\left(time6\right)+\beta_{2}*\left(time12\right)+\beta_{3}*\left(intervention*time6\right)+\beta_{4}*\left(intervention*time12\right)$$

We will estimate the parameters in the model using the SAS procedure MIXED (Cary, NC). This method handles dropout in a principled manner. However, depending on the type and scope of any missing data, we will also explore multiple imputation as a strategy to use in conjunction with our primary analytic tools [61]. As recommended in the Committee for Proprietary Medicinal Products guidelines, the primary (as well as secondary) analyses will include the race stratification variable as a fixed covariate in the main analytic models. Secondary analyses will be conducted in a similar manner, testing for differences in physical function and depressive symptoms.

For the Duke-based study, our two primary hypotheses will also be tested using a similar hierarchical linear model, with patients nested within clinic. The predictors in this model will include a dummy coded time effect and separate indicator variables for the Provider and Patient interventions interacting with the time effect. The fixed-effect portion of the model will have the form

(2) $$\begin{array}{l} Y_{\mathrm{ijk}}=\beta_{0}+\beta_{1}*\left(time6\right)+\beta_{2}*\left(time12\right)+\beta_{3}*\left(provider*time6\right)+\beta_{4}*\left(patient*time6\right)\\ +\beta_{5}*\left(provider*patient*time6\right)+\beta_{6}*\left(provider*time12\right)+\beta_{7}*\left(patient*time12\right)\\ +\beta_{8}*\left(provider*patient*time12\right) \end{array}$$

for clinic *i*, patient *j*, at time *k*.

The specific test for H_1_ based on the above model parameterization is testing that *β*_*8*_ equals zero. For H_2_, for the Provider intervention alone compared to usual care at 12-months is a test of *β*_*6*_ equal 0 and for the Patient intervention only it is *β*_*7*_ equal zero*.* The primary (as well as secondary) analyses will include the race and gender stratification variable as a fixed covariate in the main analytic models. Secondary analyses will be conducted in a similar manner, testing for differences in physical function and depressive symptoms.

### Economic evaluation

The objectives of the economic evaluation are to: 1) estimate the cost per participant for each study group; 2) estimate the annual OA-related healthcare utilization cost per participant in each group; 3) estimate the effectiveness achieved by each intervention; 4) estimate the incremental cost-effectiveness of each intervention. The Patient intervention cost will consist of labor costs (e.g., counselor training and telephone calls) and equipment and materials costs. The incremental cost incurred for the Provider intervention is primarily due to the additional time needed to collect the information from patients. Based on our prior experience with these assessments, we estimate it will require 15 minutes of time. In clinical practice, these measures would likely be obtained by a nurse. The application of per-minute wage rates will be used to derive this nurse cost.

We expect the interventions may affect two types of healthcare utilization: outpatient visits (including both primary care and specialist visits for OA-related care) and pain medication use. We will collect outpatient visit data primarily from the VA’s Decision Support System administrative dataset and Duke University Medical Center billing data. However, we will also ask patients to report office visits outside of these healthcare systems in follow-up surveys. For the VA –based study, pain medication use will also be extracted from the Decision Support System. We will also ask patients in both VA-based and Duke-based studies to report their pain medication use. For standardization, consistency, and to best approximate cost (rather than charges or reimbursement), we will use Medicare reimbursement rates to monetize outpatient visits. Market prices (e.g., from drugstore.com) will be applied to the reported medications to derive medication cost.

We will use three effectiveness measures to calculate cost effectiveness ratios: WOMAC units (primary outcome), pain-free days (during the prior 30 days), and quality-adjusted life years. The EuroQoL is used to conduct the utility measurements necessary to calculate quality-adjusted life years [62]. Bivariate analyses will be conducted to examine differences in costs and effectiveness among the intervention arms. We will then calculate the incremental cost effectiveness ratio (ICER) of the intervention arms and control group relative to each other. The ICER will be calculated as the difference in the average cost per participant divided by the difference in the average effectiveness per participant between study group.

### Timeline

Recruitment of participants began in August, 2011 and July, 2011 for VA-based and Duke -based studies, respectively. We expect that recruitment will be completed by September, 2012 and July, 2013, respectively.

## Discussion

There are several important ways in which these studies will advance our understanding of effective interventions to improve OA outcomes. First, this is one of few studies to examine a provider-based intervention for hip and knee OA, and to our knowledge it is the first to evaluate an intervention that is feasible to disseminate broadly at relatively low cost and resource use. This provider intervention is also novel because it involves patient-specific recommendations (e.g., not only general information about OA treatment guidelines) and is delivered at the point-of care. Second, although there have been many studies of patient-based behavioral interventions for hip and knee OA, to our knowledge this is the first to combine comprehensive physical activity, weight management, and cognitive behavioral pain management interventions into a telephone-based program for patients with OA. Because each of these behaviors has clinically relevant effects on OA-related outcomes, through different mechanisms, their combination may be stronger than any one or two of these components alone. Third, these two studies provide an opportunity to examine the interventions in two different real-world clinical settings. Most prior studies of OA interventions have involved either community-based, self-referred samples (which are likely a select sub-group) or patients at academically-based medical centers (which can differ in many ways from other primary care clinics). These studies are being conducted in a VA medical center, which serves many patients with complex medical needs, and in a group of Duke community-based primary care clinics of diverse sizes, organizational structures, locations (urban vs. rural), and patient samples. At both sites, a population recruitment strategy is employed rather than relying on self-referral. These attributes will enhance generalizability of study findings and provide an excellent picture of the feasibility and effectiveness of the interventions in a variety of clinical settings.

## Competing interests

The authors declare that they have no competing interests.

## Authors’ contributions

KDA is principal investigator on the projects and leads the trials. HBB, EZO, and WSY contributed to study conception, design and logistics. WSY and JM contributed to the development of weight management component of the patient intervention. JM also trains the health educators in this component of the intervention. JLS contributed to the development of the cognitive behavioral skills for managing pain component of the patient intervention and trains the health educators in part of the intervention. DSB and KAJ contributed to the development of the patient education program and deliver this program. RJD and RC contributed to the development and logistics of the provider intervention. CJC and ASJ developed the plans for statistical analysis of the studies. NS contributed to development of study measures, particularly related to qualitative participant interviews. SKD developed the health economic analysis plan for the study. JGC and CS contributed to development of overall study logistics and coordinate the projects. JK, LEM, and MPS assisted with development of processes for participant screening, recruitment, and outcome assessment. All authors critically reviewed the manuscript and approved the final version.

## Pre-publication history

The pre-publication history for this paper can be accessed here:

http://www.biomedcentral.com/1471-2474/13/60/prepub

## Supplementary Material

Additional file 1 Appendix I: Provider Intervention Recommendations and Criteria Click here for file
