# Supplementary material for: Patient and provider interventions for managing osteoarthritis in primary care: protocols for two randomized controlled trials
Source: BMC Musculoskelet Disord. 2012 Apr 24;13:60. doi: 10.1186/1471-2474-13-60 (PMC3433311; doi:10.1186/1471-2474-13-60)
Supplement: Additional file 1 — Appendix I: Provider Intervention Recommendations and Criteria [file 1471-2474-13-60-S1.doc]

|  |  |
| --- | --- |
|  |

**Appendix I: Provider Intervention Recommendations and Criteria**

**Refer to physical therapy for evaluation and/or therapeutic exercises**

Criteria:

- Patient may be interested in being referred for physical therapy for OA if their provider recommends, AND
- Patient is not doing lower extremity strengthening exercises ≥2 times per week, AND
- Patient indicates being dissatisfied with their ability to perform one more activities on the Satisfaction with Physical Function Scale (walking, lifting / carrying, stair climbing, housework), AND
- Patient has not seen a physical therapist for their OA in the past year.

**Refer for evaluation for knee brace**

Criteria: (for each knee with OA):

- Patient is not currently using a knee brace, AND
- Patient may be interested in trying a knee brace (or different kind of knee brace) if their provider recommends.

Criteria for Specific Brace Consults (VA-Based Study Only)

- Knee Sleeve: Knee pain rating 1-3 (on a 10cm visual analog scale) AND varus/valgus alignment <10°, AND does not indicate knee “buckling”.
- Hinged Brace: Knee pain rating >3 (on a 10cm visual analog scale) OR indicates knee “buckling”, AND varus/valgus alignment ≤15°.
- Unloader Brace: Knee pain >3 (on a 10 cm visual analog scale) OR indicates knee “buckling,” AND varus/valgus alignment >15°.

**Refer to** **weight management** **program**

Criteria:

- Patient has BMI ≥ 25, AND
- Patient may be interested in being referred to a weight management program if their provider recommends.

**Refer to physical activity program**

Criteria:

- Patient is not doing at least 2 hours and 30 minutes of aerobic activity per week and strengthening exercises ≥2 times per week, AND
- Patient may be interested in being referred to a physical activity program if their provider recommends.

**Perform or refer for** **Intra-articular injection**

Criteria:

- Patient has moderate to severe knee pain (≥6 on a 10cm visual analog scale), AND
- Patient has radiographic evidence of OA in that knee, AND
- Patient is already taking oral pain medications, AND
- Patient has not received a joint injection in the past 6 months, AND
- Patient may be interested in having a knee joint injection if their provider recommends.

**Recommend or prescribe** **Topical NSAID or capsaicin**

Criteria:

- Patient is not currently using topical creams for OA, AND
- Patient may be interested in trying a topical cream (or different type of topical cream) if their provider recommends.

**Patient reports taking an NSAID (prescription or OTC) but has risk factors for GI bleeding. Consider addition of gastroprotective agent or switch to other pain medication.**

Criteria:

- Patient is currently using an NSAID without gastroprotective agent, AND
- Patient has one or more risk factors for GI bleeding: age≥ 75 years, history of peptic ulcer disease or GI bleeding, current glucocorticoid use.

**Discuss the possibility of trying a new/alternate pain medication with patient**

Criteria:

- Patient indicated they may like to talk with their health care provider about the possibility of trying a different pain medication for their arthritis.

**Referral to orthopedics for evaluation for joint replacement surgery (if no contraindications to surgery)**

Criteria:

- Radiographic evidence of OA in that joint, AND
- Patient has tried each of the following: pain medications, joint injection, physical therapy, AND
- Pain ≥6 (on a 10cm visual analog scale) in that joint, AND
- Functional limitation due to OA ≥6 (on a 10 point visual numeric scale), AND
- Patient indicated they may be interested in being referred to a specialist for evaluation for potential joint replacement surgery.
